# Spatial and deep learning analyses of urban recovery from the impacts of COVID-19

**DOI:** 10.1038/s41598-023-29189-5

**Published:** 2023-02-11

**Authors:** Shuang Ma, Shuangjin Li, Junyi Zhang

**Affiliations:** 1 College of Civil Engineering and Architecture, Zhejiang University, Hangzhou, 310058 China; 2 Graduate School of Advanced Science and Engineering, Hiroshima University, Higashihiroshima, 739-8529 Japan; 3 School of Transportation, Southeast University, Nanjing, 211189 China; 4 Graduate School of Advanced Science and Engineering, Hiroshima University, Higashihiroshima, 739-8529 Japan

**Keywords:** Infectious diseases, Socioeconomic scenarios, Sustainability

## Abstract

This study investigates urban recovery from the COVID-19 pandemic by focusing on three main types of working, commercial, and night-life activities and associating them with land use and inherent socio-economic patterns as well as points of interests (POIs). Massive multi-source and multi-scale data include mobile phone signaling data (500 m × 500 m), aerial images (0.49 m × 0.49 m), night light satellite data (500 m × 500 m), land use data (street-block), and POIs data. Methods of convolutional neural network, guided gradient-weighted class activation mapping, bivariate local indicator of spatial association, Elbow and K-means are jointly applied. It is found that the recovery in central areas was slower than in suburbs, especially in terms of working and night-life activities, showing a donut-shaped spatial pattern. Residential areas with mixed land uses seem more resilient to the pandemic shock. More than 60% of open spaces are highly associated with recovery in areas with high-level pre-pandemic social-economic activities. POIs of sports and recreation are crucial to the recovery in all areas, while POIs of transportation and science/culture are also important to the recovery in many areas. Policy implications are discussed from perspectives of open spaces, public facilities, neighborhood units, spatial structures, and anchoring roles of POIs.

## Introduction

The global confirmed infection cases of COVID-19 have increased more rapidly since the beginning of the year 2022 than 2020 and 2021. There are many factors contributing to the COVID-19 spread, such as social contact^1–4^ and violation of social distancing rules^5,6^, built environment and transportation factors^7–9^, socio-economic factors^10^, and meteorological factors^11^. There is further direct evidence showing a strong correlation between the number of coronaviruses (including SARS-CoV-2) and local bat species richness (on average, 2.7 coronaviruses harbored by each bat species) in Yunnan province of China and neighboring regions in Myanmar and Laos, which are shown to form a global hotspot of climate change-driven increase in bat richness^12^. Many countries over the world have been struggling to figure out how to ensure a robust recovery from this pandemic, where cities are at the core of the recovery due to various reasons (e.g., difficulty to control the virus spread, the engine of economic development, and more concerns about social exclusion). Urban recovery reflects the ability of a city to withstand shocks and stresses^13^, while the urban recovery from COVID-19 should be investigated by reflecting the influences of multiple factors that affect the COVID-19 spread and are associated with economic activities. The urban recovery from COVID-19 is also crucial to the human sustainability^14^. Understanding relationships between human mobility and recovery is important to the promotion of effective urban recovery which is in line with sustainable development^15^. Furthermore, global warming has also led to melting glaciers, which could unleash ancient viruses^16^. As stated by Zhang and Hayashi^17^, “mankind may be continuously threatened by viruses. Therefore, the human society has to equip all human systems to combat the spread of future viruses”. However, existing research efforts of exploring urban recovery from COVID-19 and its associated factors are still very limited^18,19^.

Therefore, more scientifically sound insights into recovery policymaking should be accumulated for not only the fight against the ongoing pandemic but also the post-pandemic urban transformation. This research attempts to provide additional evidence from a perspective of human mobility for the urban recovery from COVID-19, especially by investigating the following unresolved research issues.

(1) Limited evidence of urban recovery from perspectives of multi-faceted aspects of human mobility.

Liu et al. revealed uneven recoveries across cities after lockdown in China by using population flow data from 327 cities from Gaode Map based on a difference-in-differences model and found that the level of human mobility could return to the before-pandemic level, 6 weeks after relaxation strategies in most cities were released and 12 weeks for cities with the worst infections^20^. By applying a machine learning approach to analyze mobile phone signal data in Seoul metropolitan area, South Korea from 2020 (between February 17 and May 29) and 2019 (between February 18 and May 31), Eom et al. found that more employees of real estate, public administration and health care were associated with a higher-level recovery measured at the level of traffic analysis zone^21^. Using mobile phone network data in Beijing, Dong et al. confirmed that areas with large-scale parks and areas far from the city center recovered fasters than other areas^22^. Gutiérrez et al. discussed the various difficulties of the recovery of public transport ridership and the importance of constructing sustainable and human-scale^23^. Buffel et al. reviewed the various unequal impacts of COVID-19 in terms of age, gender, social class, ethnicity, race, ability and sexuality and based on such reviews, they further proposed six principles for ‘age-friendly’ community recovery planning from perspectives of resource allocation, narrative on aging, age inclusivity, investment in community-level services and built environment, local partnership, and involvement of older people^24^. Based on extensive literature reviews, Moglia et al. summarized lessons learned from COVID-19 for outlining urban missions for a green urban recovery and defined six pathways that support urban transitions in terms of mobility, energy, food, housing, health, and nature^25^; however, they did not make any empirical analysis from such multiple perspectives. As revealed above, evidence about the urban recovery from COVID-19 is still limited from perspectives of multi-faceted aspects of human mobility related to various levels of production, leisure, and consumption.

(2) Little evidence related to spatially unequal urban recovery at fine-grained level.

Urban recovery is the ability to recover from disruptions^26,27^. Such an ability may not be the same across various areas in a city. Unequal recovery may lead areas with low recovery ability losing their competitions. It could create new unbalanced and unstable social and economic development for a city. There are many studies investigating the relationships between human mobility and COVID-19 spread as well as pandemic policymaking with respect to initial stages or accelerating stages of the pandemic. Such analyses are usually done at relatively large spatial scales, regarding the effectiveness of pandemic policies^28–30^. It is further found that the human activity recovery from COVID-19 was more efficient in metropolitan areas than in rural areas^31^, uneven urban recovery was affected by the pandemic severity^20^, and higher urban recovery was observed at those neighborhoods with low-density and older population^32^. However, existing studies have neglected the exploration of spatially unequal recovery at the fine-grained level that allows policymakers to make more spatially focused and publicly acceptable policy measures.

(3) Little evidence about influential urban functions at fine-grained level and influences of pre-pandemic socio-economic activity patterns.

Even though scholars such as Deas et al.^33^ explored the temporary reuse of land and buildings in response to the COVID-19 crisis to improve urban resilience, their suggestions only have short-term experimental values. It is observed that the spread of COVID-19 associated with the built environment is affected by their neighboring townships and cities^8,9^. Dong et al.^22^ conducted a recovery analysis of Beijing at a spatial scale of 1.0 km × 1.0 km. Related to Nagoya, socioeconomic factors and land use patterns at the zip-code level are revealed to influence the frequency of visiting green infrastructures during the COVID-19^34^; using mobile spatial statistics (with a minimal mesh of 1.0 km × 1.0 km) to evaluate the characteristics of population at different stages of COVID-19, it is found that population recovery is more robust at Nagoya Station and Sakae on weekdays and holidays^35^ (note that in Nagoya, its central area was the most visited place before the pandemic^36,37^, similar to other cities in Japan; but no study had not been done on the influences of pre-pandemic social-economic activity patterns on urban recovery). Similar cross-space associations may exist in the urban recovery that needs to be realized over wider areas; however, little has been done from such a perspective. People resume their activities that are usually performed at specific facilities with suitable services. However, the urban recovery from COVID-19 has not been explored at the level of facility. Furthermore, people’s behaviors are habitual in many cases. Similarly, many economic activities are also habitual.

### Research purpose

This research attempts to fill the aforementioned research gaps with an aim at not only providing scientific insights into the urban recovery policymaking and post-pandemic long-term strategies but also advancing pandemic research with significant added values in the urban context to the literature based on methodologically sound data-driven approaches. Targeting the whole Nagoya of Japan, a period after lifting the declaration of a state of emergency over the whole country (note: in Japan, declaring a state of emergency is the strictest pandemic-control measure) was selected and compared with the same period in 2019.

### Research contents and methodologies

Keeping the above aim in mind, first, this study reveals spatially unequal recoveries of three key urban activities (i.e., working, commercial and nigh-life activities) by using mobile phone signaling data in the first recovery period of 2020 at a 500 m × 500 m mesh level (1209 meshes covering the whole Nagoya), in comparison to the same period in 2019. Five levels of urban recovery (from 1 (lowest) to 5 (highest)) are classified for revealing nonlinearities existing in both the recovery degree and its associated factors for considering both modeling accuracy and convenience of exploring the urban recovery. The following deep learning approaches further make the best use of such categorized variables. Second, a convolutional neural network (CNN) model is applied to learn/predict the urban recovery level with the 500 m × 500 m resolution by associating with visual attributes of aerial images with the 0.49 m × 0.49 m resolution, extracted through creating visual explainable layers. Third, to quantify the importance of the hidden and complex associations between aerial images and the urban recovery levels, detected from the CNN model, one more deep learning approach “Guided Grad-CAM model” is adopted by generating activations maps that are further spatially combined with land use patterns. Fourth, the above-generated activation maps are then overlayed with land use and pre-pandemic socio-economic activity patterns at the street-block level (in total, 4086 blocks) in the GIS platform (ArcGIS Pro), and using activated rates and mean activated values, spatial clusters/outliers of the fine-grained urban recovery are identified by focusing on connections across spatial neighbors with the assistance of Bivariate Local Indicator of Spatial Association (BiLISA) approach through ArcGIS Pro. Pre-pandemic socio-economic activity indicators are measured using night light satellite data at the approximate 500 m × 500 m mesh level, which are merged into the street-block level when clustering the above-mentioned land use and activity patterns based on Elbow method and K-means method. Fifth, the most influential land use and inherent socio-economic activity patterns are derived by comparing the average activated rates and mean activated values respectively, through overlaying the use and inherent socio-economic activity patterns with activation maps. Finally, the influential POIs (from 39,761 POIs from 263 categories) are identified by overlaying the spatial cluster/outlier layers, activation map and POIs layer in ArcGIS Pro.

## Results

### Spatial distributions of urban recovery levels

**Figure 1:**
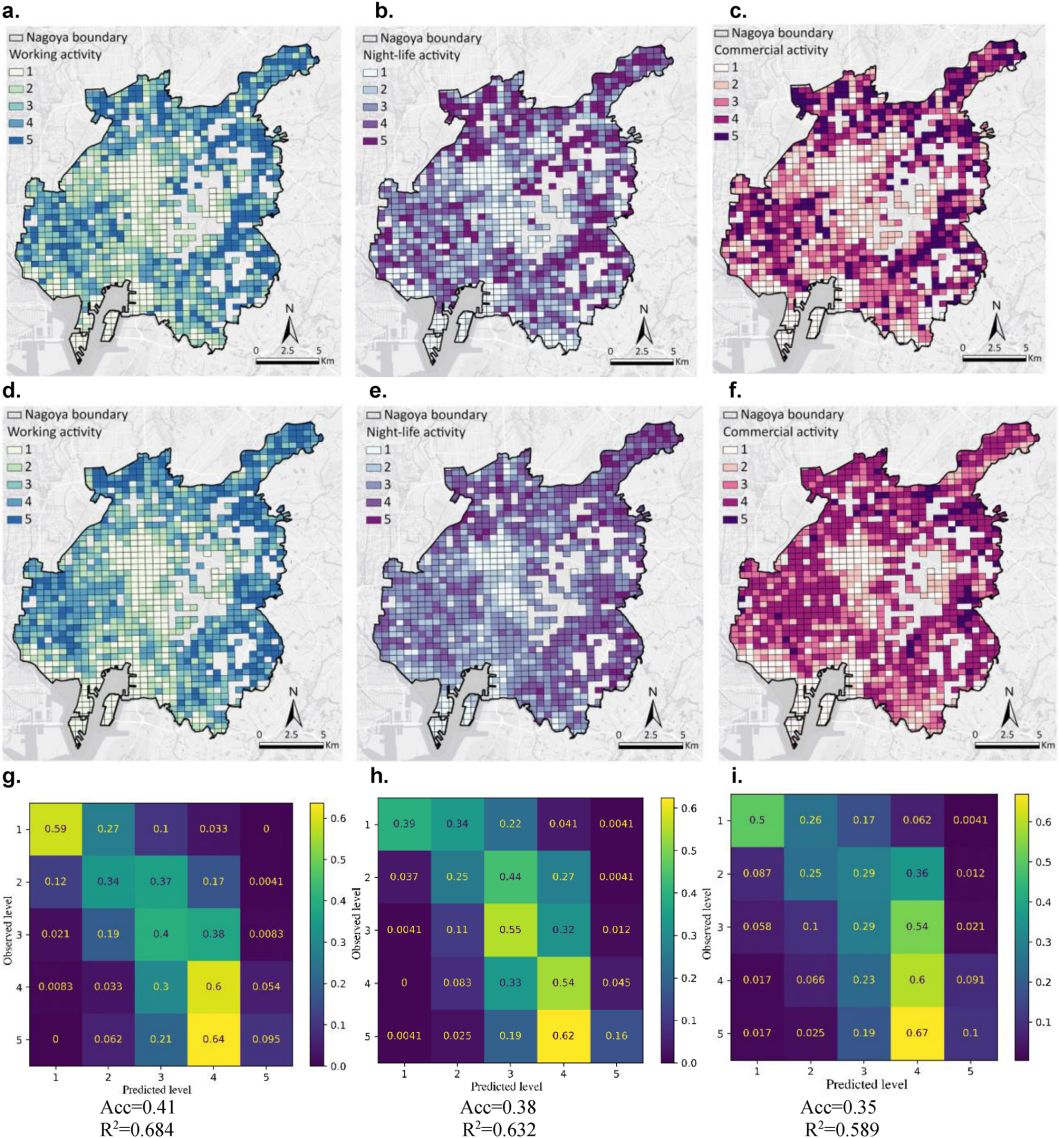
Observed and predicted urban recovery levels in Nagoya. (**a**–**c**) Observed urban recovery levels for working, night-life, and commercial activities, respectively; created using ArcGIS Pro Version 2.9 from ESRI (http://www.arcgis.com/). (**d**–**f**) Predicted urban recovery levels for working, night-life, and commercial activities, respectively; created using ArcGIS Pro Version 2.9 from ESRI (http://www.arcgis.com/). (**g**–**i**) Hit rates for working, night-life, and commercial activities, respectively; created by Anaconda Navigator Version 1.9.12 from Anaconda (https://www.anaconda.com).

Figure 1a–c illustrates the spatial distributions of the five recovery levels with respect to the three selected main urban activities, measured by mobile phone signaling data. On average, the recovery level of working activities is the highest, followed by night-life and commercial activities. This observation indicates that the recovery from the current pandemic may be first driven by the working activities. This seems understandable, considering the essential features of working activities as a key part of economic activities. Once the working activities are resumed, they will generate more needs of commercial activities. Thus, the second largest recovery level of commercial activities seems intuitive. On the other hand, the night-life activities are performed by not only general citizens who want to relax through eating out and drinking, but also working persons who need to communicate with their colleagues and/or business partners through eating out with drinking during the night time. Concerning spatial distributions of the recovery, lower recovery levels are mainly observed at central areas in Nagoya, and higher recovery levels appear at peripheral areas of the city center and suburbs. Thus, the recovery in Nagoya shows a doughnut distribution. Using detailed mobile phone signaling data covering a whole large-sized city, such doughnut-shaped recovery is one of the initial observations in the literature. More details can be found at the Section 2A in Supplementary Information.

### The fine-grained associations of land use and inherent socio-economic patterns and urban services with urban recovery

#### Estimating and validating an EfficientNetB0-based urban recovery prediction model

Figure 1g–i shows how well the EfficientNetB0 model predicts the five urban recovery levels corresponding to the three selected urban activities, where the diagonal cells of those matrices indicate perfect match between observed recovery levels and predicted ones (the upper three matrices show the number of 500 m × 500 m meshes and the lower three present the percentages) (more explanations of the analysis accuracy refer to the Section 2B in Supplementary Information). Figure 1d–f show how well the prediction model can predict urban recovery levels. Comparing Fig. 1a–c and g–i, it is found that the built EfficientNetB0-based urban recovery prediction model presents very similar results to actual recovery levels with respect to all the three selected urban activities.

#### Associating aerial images and “land use and pre-pandemic socio-economic activity” patterns with urban recovery through activation maps

**Figure 2:**
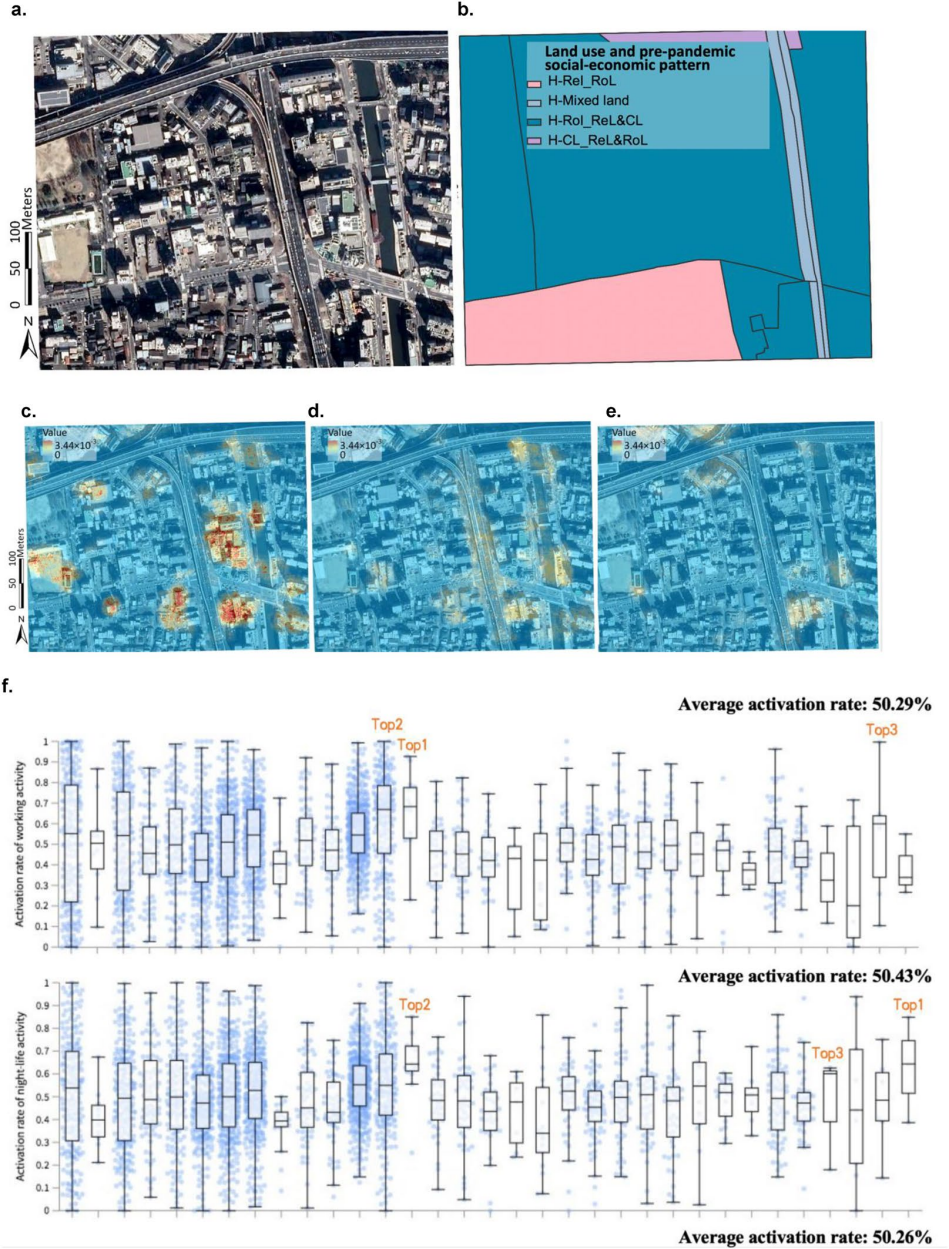

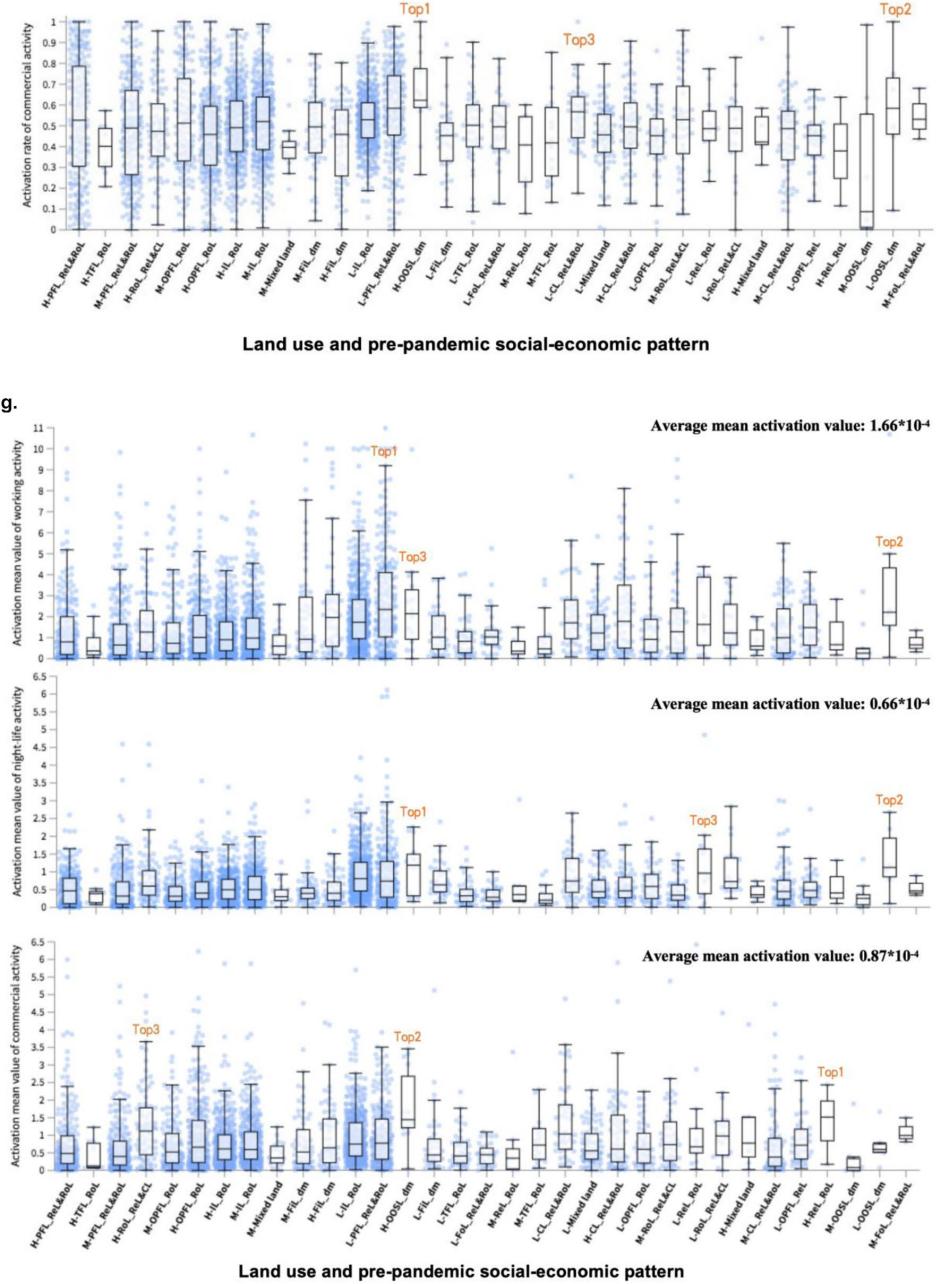
Interpretable activation map in an example mesh and activation rates and activation mean values for three different activity sectors in Nagoya. (**a**) Aerial image in an example mesh; obtained from Google static maps (https://earth.google.com/web) and mapped using ArcGIS Pro Version 2.9 from ESRI (http://www.arcgis.com/). (**b**) Urban function in street-blocks in the example mesh. (**c**–**e**) Generated from the aerial image (**a**) by using guided Grad-CAM to compute activation maps for working activity (**c**), night-life activity (**d**), and commercial activity (**e**). (**f**,**g**) Activation rates (**f**) and activation mean values (**g**) in different “land use and pre-pandemic socio-economic activity” patterns for three different activity sectors in Nagaya. Note: L, M, and H refer to low-level socio-economic activity, medium-level socio-economic activity, and high-level socio-economic activity, respectively; Unit for activation mean value is 10^−4^; *IL_RoL* Industrial lands with road lands, *PFL_ReL&RoL* public facility lands with residential lands and road lands, *FoL_ReL&RoL* forest lands with residential lands and road lands, *OPFL_ReL* other public facility lands with residential lands, *FiL_dm* field lands dominated, *RoL_ReL&CL* road lands with residential lands and commercial lands, *ReL_RoL* residential lands and road lands, *TFL_RoL* transportation facility land with road lands, *OPFL_RoL* other public facility land with road lands, *CL_ReL&RoL* commercial lands with residential lands and road lands, *OOSL_dm* other open space lands dominated.

Urban recovery levels, land use and socio-economic patterns, and aerial images are available at the 500 m × 500 m mesh level (1209 meshes), the street-block level (4086 blocks), and the 0.49 m × 0.49 m pixel level, respectively. To connect aerial images with the urban recovery levels in an interpretable way, activation maps were created by Guided Grad-CAM. Figure 2 shows examples of how the associations of aerial images and “land use and socio-economic activity” patterns with urban recovery are derived from the analysis. The examples in Fig. 2a,b illustrate aerial images and “land use and socio-economic” patterns in each street-block. Aerial images were obtained from Google static maps (https://earth.google.com/web) and loaded in Python for training and inferring. After dividing land uses related to three pre-pandemic social-economic activity levels based on the night-time light data, the social-economic activity attributes were added in the land use layer and further visualized as shown in the example of Fig. 2b in ArcGIS Pro. As shown in Fig. 2c–e, the interpretable features generated by Guided Grad-CAM are overlaid to the aerial image map in ArcGIS Pro. The highlighted area (with a 0.49 m × 0.49 m resolution) in the activation map visualizes the most important features associated with urban recovery measured in terms of the three selected urban activities. More details are available at the Section 2C in Supplementary Information.

#### The street-block-level associations between activation values with “land use and pre-pandemic socio-economic activity” patterns

Figure 2f displays the street-block-level associations between activation rates (activation mean values) and the 33 “land use and pre-pandemic socio-economic activity” patterns in Nagaya. These patterns were generated by combining 12 land use patterns (details refer to the Section 3H in Supplementary Information) derived from Elbow and K-means method and the three levels (High: H, Medium: M, and Low: L) of pre-pandemic socio-economic activities detected by the night-time light data, where there were three missing combinations. For the three activities, their average values of activation rates are similar (50.29%, 50.43% and 50.26%, respectively) with respect to all the 33 patterns. For working activities, the highest correlation is observed in H-OOSL_dm (68%), L-PFL_ReL&RoL (67%), and L-OOSL_dm (60%). For night-life activities, the top three important patterns to represent activation rates are the M-FoL_ReL&RoL (64%), H-OOSL_dm (64%) and H-ReL&RoL (60%). With regards to commercial activities, the H-OOSL_dm (62%) is the most important pattern, followed by the L-OOSL_dm (58%) and the L-CL_ReL&RoL (57%). The distance between 25th percentile and 75th percentile at H-OOSL_dm is smaller for night-life activities, meaning that the influences of this patten across street blocks are similar. With regards to working activities, 69.7% blocks have at least one activated pixel (0.49 m × 0.49 m) and the corresponding percentages for night-life activities and commercial activities are 81.8% and 78.8%, respectively. The H-OOSL_dm (occupy 7.14%) for working activities, M-OOSL_dm (occupy 11.11%) for night-life activities and Mixed land (occupy 5.56%) for commercial activities separately have the highest percentage of street-blocks without activated 0.49 × 0.49 m pixels.

In terms of mean activation values (Fig. 2g), the average mean activation values are 1.66 × 10^−4^, 0.66 × 10^−4^, and 0.87 × 10^−4^ respectively for working activities, night-life activities, and commercial activities with respect to all the 33 patterns. Looking at the mean activation value in each activity sector, the L-PFL_ReL&RoL (2.34 × 10^−4^), L-OOSL_dm (2.21 × 10^−4^) and H-OOSL_dm (2.14 × 10^−4^) are highly correlated with the urban recovery in terms of working activities, H-OOSL_dm (1.19 × 10^−4^), L-OOSL_dm (1.12 × 10^−4^) and L-ReL&RoL (0.96 × 10^−4^) are highly associated with the urban recovery in night-life activities, and H-ReL&RoL (1.52 × 10^−4^), H-OOSL_dm (1.45 × 10^−4^), and H-RoL_ReL&CL (1.12 × 10^−4^) are most important to the urban recovery of commercial activities. In these land use and socio-economic patterns, the highly associated pixels play crucial roles in promoting the recovery. These results also indicate that H-OOSL_dm is one of the most correlated land use and socio-economic patterns to both variables that represent associations of urban functions with the recovery in all activities.

#### Spatial clusters of urban functions associated with urban recovery

**Figure 3:**
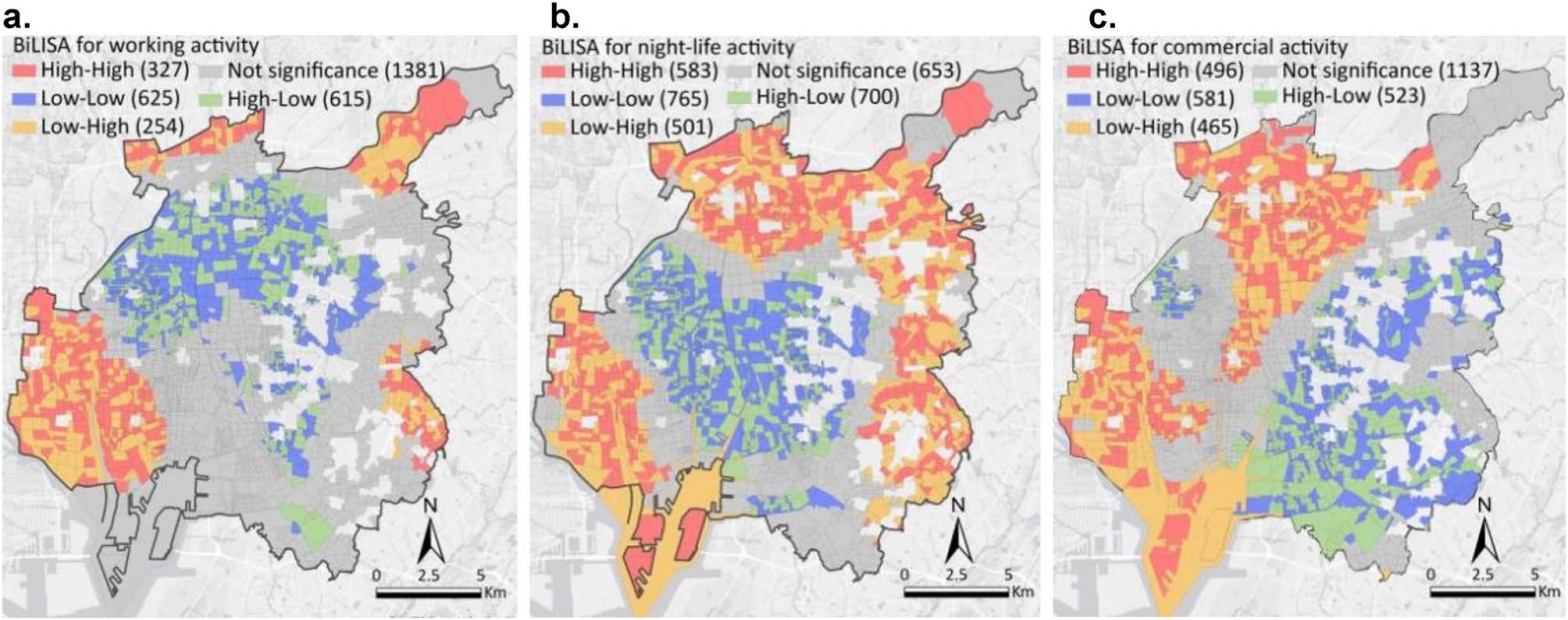
BiLISA between activation rates and activation mean values in Nagoya. (**a**–**c**) The BiLISA results for working activity (**a**), night-life activity (**b**), and commercial activity (**c**); created using ArcGIS Pro Version 2.9 from ESRI (http://www.arcgis.com/).

Figure 3 illustrates spatial clusters detected by the BiLISA with respect to activation rates and mean activation values, which show important associations of urban functions with urban recovery. The High-High type means that a street-block with a high activation rate is surrounded by neighboring street-blocks with high mean activation values, while the High-Low type shows that a street-block with a high activation rate is surrounded by neighboring street-blocks with low mean activation values. Similarly, the Low-high type indicates that a street-block with a low activation rate is surrounded by neighboring street-blocks with high mean activation values, while the Low-Low type represents that a street-block with a low activation rate is surrounded by neighboring street-blocks with low mean activation values. Here, “high” or “low” is defined relative to the mean value of each variable.

Related to working activities, the High-High and Low-High types are mainly distributed in the peripheral areas of Nagoya’s central areas in four directions: north-east, north-west, south-east, and south-west. The Low-Low and High-Low street-blocks are situated in central areas. For night-life activities, the location of each type is similar to that of working activities; however, there are more Low-High and High-High types at the periphery areas. Concerning commercial activities, there is an invisible geographic line from north-east to south-west, dividing Nagoya into two parts. The High-high type and Low-high type are located at the west, while most of the Low-low type and High-low type are agglomerated at the east.

The urban recovery degree in Nagoya has a donut distribution with more street-blocks with higher recovery located at peripheral areas. Thus, for working activities and commercial activities, the High-high type and Low-high type that are situated at peripheral areas are more essential to promote urban recovery. In other words, high activation mean values at surrounding blocks play crucial roles in urban recovery. Urban planning lessons can also be learned from other types, such as Low-low type, in which the functions at the limited activated pixels can be very important to support urban recovery. Therefore, the detailed POIs in these activated meshes at each cluster are further investigated.

#### Important POIs associated with urban recovery

**Figure 4:**
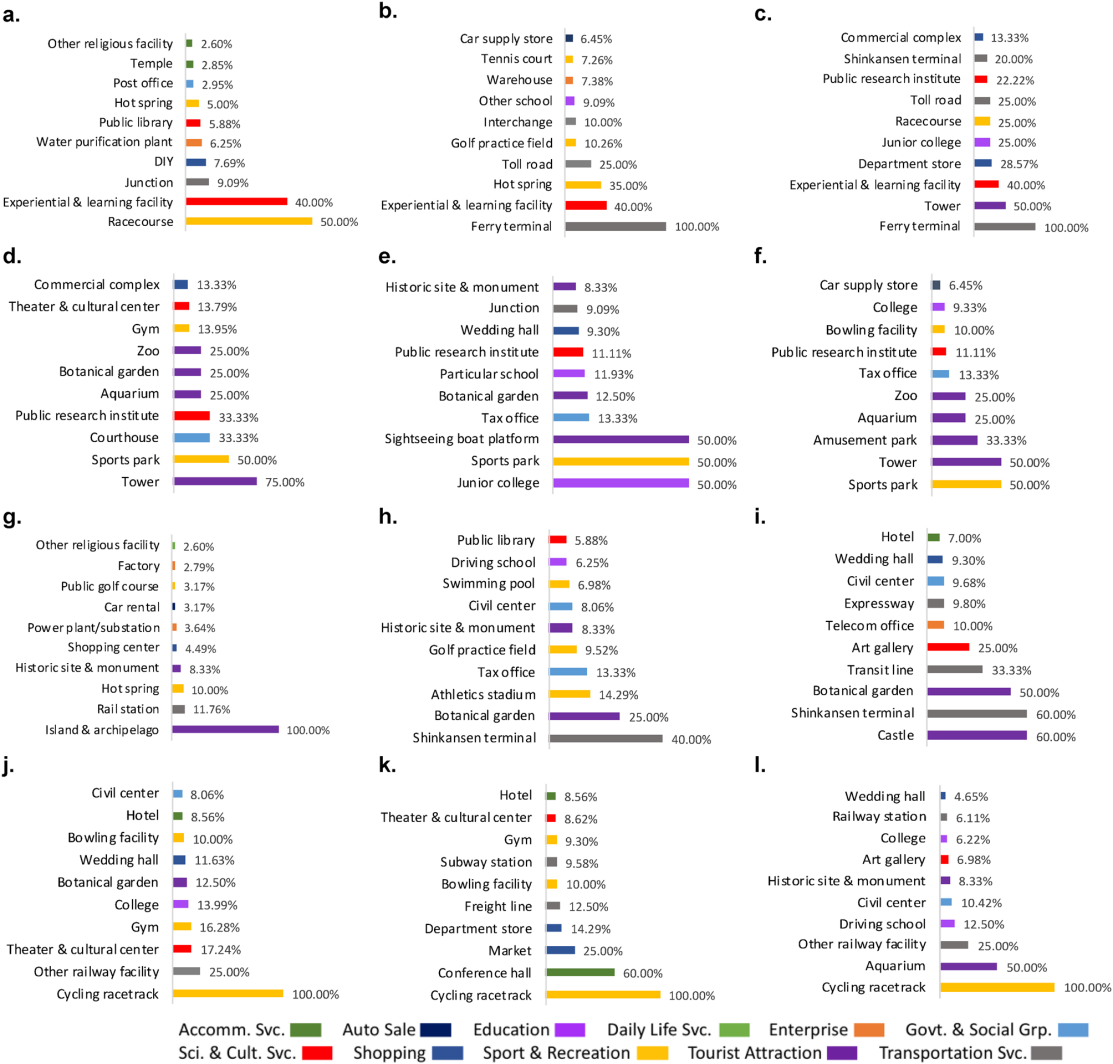
The top-10 most important POIs contributing to the urban recovery from COVID-19. (**a**–**c**) High-high type for working activity (**a**), night-life activity (**b**), and commercial activity (**c**). (**d**–**f**) Low-low type for working activity (**d**), night-life activity (**e**), and commercial activity (**f**). (**g**–**i**) Low-high type for working activity (**g**), night-life activity (**h**), and commercial activity (**i**). (**j**–**l**) High-low type for working activity (**j**), night-life activity (**k**), and commercial activity (**l**). Note: The value corresponding to each POI is the percentage of the POIs in its main category.

After overlaying the POIs layer with spatial clusters/outliers and activation maps (only with activation values being the top one-third of all values), important POIs were explored by calculating the proportion of each POI category in each type of spatial clusters/outliers, occupied the total number of each POI category in Nagoya was computed. Concretely, more important POIs were selected based on the proportions. Figure 4a–l shows the top 10 important POIs within the 16 POI categories for the three activities: working activity, night-life activity, and commercial activity.

Figure 4a–c illustrates the High-high types for the three activities. The three categories of sport and recreation, science and culture service, and transportation service are crucial. Concretely speaking, racecourses in the category of sport and recreation and experiential learning institutes in the category of science/culture service are much more important for the recovery of the working activity than other POIs in the sense that these two POIs account for 50% and 40%, respectively, within each POI category, while the shares of other POIs are just less than 10%. As for the night-life activity, three POIs belong to the category of sport and recreation and another three POIs are included in the category of transportation service, suggesting that these two POIs categories are crucial to the recovery of night-life activity. The top three POIs are ferry terminals (100%), experiential and learning facilities (40%), and hot spring (35%). For the recovery of commercial activity, ferry terminals (100%) in the category of transportations service and towers (50%) in the category of tourism attraction are much more important than other POIs. Different from the working activities, there are more POIs showing larger importance, including department stores (28.57%), junior colleges (25%), racecourses (25%), public research institutes (22.22%) and Shinkansen terminal (20%).

For the Low-low types (Fig. 4d–f), the POI categories of tourist attraction and sport and recreation are highly correlated with the urban recovery. Sports parks (50% for all in these three activities) are highly associated with the recovery from perspectives of all the three activities. Towers are ranked as the top one for the working activity and the top two for the commercial activity. Sightseeing boat platforms and junior colleges are equally important as sport parks for the night-life activity. The other POIs with larger percentages are public research institutes, aquariums, botanical gardens, and zoos.

Concerning the Low-high type (Fig. 4g–i), the categories of tourist attraction and transportation service are much more important to the urban recovery than other POI categories. Shinkansen terminal and botanical gardens are the top two POIs for night-life and commercial activities, while island and archipelago for the working activity, athletics stadiums for the nigh-life activity, and castles and art galleries for the commercial activities are also highly important to the urban recovery.

In the case of the High-low type (Fig. 4j–l), the category of sport and recreation (i.e., the cycling racetrack POI (100%)) is most important to the urban recovery. Conference halls for the nigh-life activity and aquariums for the commercial activity are also highly important because their percentage values are 60% and 50%, respectively. Other railway facilities (e.g., tram base and cargo transfer area) are further identified to be more important for working and commercial activities.

## Discussion

Motivated by the various impacts caused by the ongoing COVID-19 pandemic and recognizing other potentially emerging public health threats in future, this study has investigated the urban recovery from COVID-19 in Nagoya, Japan from perspectives of human mobility at smaller spatial scales, multi-source massive data (mobile phone signaling, aerial images, GIS data, night light satellite data), and deep learning analyses. Multi-source data and multiple deep learning models have been successfully combined to capture the uneven recovery over the whole Nagoya and its associated factors.

### Differences of this study from existing studies

The most significant difference of the current study from existing studies is the way of using aerial images. Aerial images have been widely used to examine urban land cover^38^, urban building texture^39^, rural–urban expansion^40^, and urban socioeconomic status inference^41^. However, such open-source images, especially with 0.49 m × 0.49 m resolution, had never been used to examine the urban recovery from COVID-19, even though extremely valuable information for urban planning and management is included. This study makes use of the benefits of high-resolution aerial images covering the whole area of a city and further applies deep learning methods to generate activation maps that are overlayed with various urban elements (including but not limited to urban land and facilities). Such analyses allow us to quantify the various associations of virtual features of aerial images with the urban recovery. In addition, no study has been done to examine the urban recovery from COVID-19 based on phone signaling data with 500 m × 500 m resolution covering the whole area of a city, by targeting main urban activities related to working (linked with employment), commercial activities (i.e., business), and night-life activities (i.e., discretionary activities). Furthermore, no study can be found to derive important urban facilities/services for the urban recovery by connecting with POIs and pre-pandemic socio-economic activities. Last but not the least, this research can be well differentiated from existing studies in terms of the joint use of CNN and Guided Grad-CAM, Elbow and K-means as well as BiLISA, which are suitable to deal with massive multi-source and multi-scale data, simultaneously.

### Importance of the current study

Urban areas with 90% reported infection cases^42^ are the key place to the recovery from COVID-19. In this regard, existing studies have argued the importance of urban design for people and nature and urban resilient infrastructures^25^, discussed the role of culture^43^, and empirically investigated the role of urban prosperity (related to pre-pandemic socio-economic activities)^44^. However, the arguments/discussion are conceptual, only based on literature review and without direct empirical evidence, and the investigation only targeted healthcare workers. For advancing research on urban recovery from COVID-19, this study has provided empirical evidence by targeting a whole city and main urban activities based on scientifically sound methodologies. Targeting Nagoya, the most important city in central Japan, empirically examining its recovery from COVID-19 has not only nationwide implications but also long-term values to many other cities with similar population sizes in the world. Making effective use of visual features contained in 0.49 m × 0.49 m aerial images supported by mixed approaches (i.e., deep learning, spatial and statistical analyses) provides a new lens of exploring spatially focused urban recovery from COVID-19 and derives more publicly acceptable insights into post-pandemic urban transformation toward a better state of sustainability. This study further confirms the importance and the various possibilities of making better use of multi-source and multi-scale data at the city level.

### Findings

The urban recovery in Nagoya is revealed to show a donut-shaped spatial pattern. Concretely speaking, the higher-level recovery is observed at peripheral/suburban areas with lower population density, rather than central areas, especially in terms of working activities, followed by night-life activity and commercial activity. More open spaces are most important to urban recovery at areas with high-level pre-pandemic social-economic activities, as observed by the influence of OOSL_dm.

Socio-economically inactive areas and public facilities mixed with residential and road lands (i.e., L-PFL-ReL&RoL) are highly correlated with the working activity recovery. Residential areas with more roads (i.e., Rel&RoL) is important to the night-life activity recovery, which is further highly associated with the areas with moderate socio-economic activities and forest lands with residential and road lands (i.e., M-FoL_Rel&RoL). L-CL_ReL&RoL (commercial lands with residential lands and road lands) and H-RoL_Rel&CL (road lands with residential lands and commercial lands) are significantly associated with the commercial activity recovery, to which the areas with high-level socio-economic activities and certain residential and road lands (i.e., H-Rel&RoL) are also influential. Those peripheral/suburban areas surrounded by neighboring areas with high activation mean values (High-High or Low-High) (corresponding to experiential and learning facility, botanical garden, and racecourse, etc.) are important for the recovery in terms of working and night-life activities.

In terms of detailed urban functions, the recovery shows obvious spatial heterogeneities. The recovery of the areas with highly influential visual attributes (High-high and High-low types) is more attributable to those POIs in the categories of sport and recreation, transportation service, and science and culture service, and, than other types of POIs. Cycling racetrack, racecourses, ferry terminals, and experiential and learning facilities are especially important. In contrast, the recovery of the areas with less influential visual attributes (Low-high and Low-low types) is more affected by those POIs of tourist attractions, and sport and recreation, where surface railway stations, sports parks, and sightseeing spots (towers, islands and archipelagos, botanical gardens, castles, sightseeing boat platforms, amusement parks) are important. POIs of sport and recreation are crucial to the urban recovery at all areas of the whole city.

### Practical implications

#### Design and smarter management of open spaces

The various distancing policy measures taken during the COVID-19 pandemic have forced people to re-find more suitable spaces for social communications and relaxations. The roles of open spaces are once again in the limelight. At least in Japan, the target country of this study, many cities lack sufficient open spaces. Our research results suggest that more open spaces are required in cities and existing open spaces should be better re-designed and smartly managed. For example, while it is imaginable, also understandable, to re-build those abandoned urban spaces into buildings for increasing economic benefits, it is worth challenging the transformation of abandoned urban spaces into open spaces that allow people to use freely. The ground floors of larger corners of buildings can be reshaped into setback areas with benches, without disturbing other parts of buildings. Similarly, rooftop spaces can also be better used through redesign and smarter management. The walkability and safety of sidewalks should be improved.

#### Enhancing the flexibility of public facilities

For making a place to be resilient, its form must be responsive to and capable of change^45^. Our analysis results reconfirm this in terms of the use of public facilities. Concretely speaking, the flexibility design of public facilities supported by proper management is crucial for allowing flexible use of public facilities during emergent events like COVID-19 and disasters. Current unbalanced recovery between central and peripheral/suburban areas suggests that more attentions should be paid to areas with higher population density after the pandemic. Introducing more public facilities in popular areas should be set as a key policy goal.

#### Re-functioning the human-scale residential areas or neighborhood units

In many senses, the COVID-19 pandemic has allowed people to re-recognize the roles of their residential areas or neighborhoods in their daily lives. Concretely speaking, the role of neighborhood unit should be re-emphasized by giving new values for supporting the resilient urban development. Working/studying from home has forced people to make use of their residential function for work/study, while such a changed working/studying style also allows people to release from long-distance travel and make more flexible use of time. Meanwhile, longer stay at residential areas generates more needs to the neighborhoods. This supports the importance of mixed land use and added urban functions/services, which allows people to perform their daily activities at human scale.

#### Re-design of the commercial functions with a better hierarchical spatial structure

The faster recovery of commercial activities at peripheral/suburban areas suggests that the commercial functions within cities must have a better spatial structure within a city, rather than the sole focus on its central area. In other words, commercial facilities with various sizes and improved transport accessibility should be properly located within different parts of a city, allowing people to meet their commercial needs without traveling a long distance and/or going to a place with a higher population density. Considering the ever-increasing popularity of online shopping during the COVID-19 pandemic, areas with commercial functions should be redesigned by accommodating consumers’ heterogeneous needs for urban spaces/functions and affective feelings during experiencing such urban spaces/functions.

#### Enhancing the anchoring roles of key POIs at various locations

Various POIs (e.g., facilities, buildings, spaces) of different types have been identified to affect the urban recovery in diverse ways. It seems that there are some key POIs that have played the anchoring roles in guiding/supporting people to survive during the COVID-19 pandemic. Among the various POIs, sports/recreation and urban tourism facilities are found to be useful for residents to re-recognize the values of their surrounding urban functions. In this special city of Nagoya, railway stations seem to be another anchor point facilitating human mobility. More anchor points (in central areas, suburban areas, etc.) should be found, through the assistance of activation maps, for supporting the post-pandemic urban transformation.

### Contributions

This study has advanced the research on urban recovery and pandemics from the following aspects.

#### Methodological contributions

Mixed data-driven approaches (deep learning and statistical/spatial analysis approaches: CNN, Guided Grad-CAM, Elbow and K-means) are jointly used to deal with massive multi-source and multi-scale data (mobile phone signaling data (mesh: 500 m × 500 m), aerial images (pixel: 0.49 m × 0.49 m), night light satellite data (approximate 500 m × 500 m), GIS-based land use data (street-block: much smaller than 500 m × 500 m), and POIs data (point: corresponding to a pixel)) for a better inference of the urban recovery mechanisms.

#### Practical contributions

Findings (especially related to unequal spatial recovery) and policy implications are derived for realizing post-pandemic transformations through strategic urban planning and design as well as land management. Use of aerial images with 0.49 m × 0.49 m resolution to explain the urban recovery level captured by spatial clusters/outliers provides spatially focused evidence with area-specific features for the redesign of urban facilities/services and mobility.

#### Long-term values

More emerging public health threats are expected to occur in future. The human society must prepare for the occurrence of similar pandemics like COVID-19. The current study has presented various findings, from a perspective of urban planning, design, and management, which have long-term values for reference.

### Limitations and future challenges

This research has some limitations. First, mobile phone signaling data with a spatial scale smaller than the 500 m × 500 m mesh should be used for a better matching with the fine-grained scale of aerial images. Second, more and longer periods without infections should be investigated to examine the robustness of the findings derived from the current study. Third, this study has attempted to capture the meanings of aerial images by overlaying with patterns of land use and pre-pandemic socio-economic activities as well as POIs, but in future, it is necessary to directly identify objects/facilities/functions from aerial images based on more innovative deep learning and spatial analysis models. Fourth, only Nagoya was selected as a case to discover the connections between urban recovery and urban functions (facilities/services) and only a special recovery period was targeted. Fifth, if data is available, more types of activities, especially, behavioral changes, should be used to evaluate urban recovery in a more convincing way. Last but not the least, international comparisons should be made for deriving more general insights into the post-pandemic urban development strategies.

## Materials and methods

### Data

This study uses five types of data: i.e., mobile phone signaling data, aerial images, land use data, night light satellite data, POIs data in Nagoya (see the Section 3A in Supplementary Information). The first two types are used for the two deep learning models of CNN and Guided Grad-CAM. The other three types are used for the clustering analysis based on Elbow and K-means methods. All these five types of data are further used for BiLISA. Complete information can be found at the Section 3B–F in Supplementary Information.

### Measurement of urban recovery

Urban recovery is measured with respect to different types of activities at the 500 m × 500 m mesh level. The level of urban recovery in terms of a certain activity type is calculated as the ratio of the activity intensity in a selected period (i.e., May 26 (Tuesday)–June 1 (Monday)) of 2020 relative to that in the same period (May 28 (Tuesday)–June 3 (Monday)) in 2019. The following three types of activities are selected: working activities, night-life activities, and commercial activities. Details can be found at the Section 3G in Supplementary Information.

### Estimating the importance of aerial images based on CNN and Guided Grad-CAM

This study not only explores urban recovery itself but also examines what kinds of land use and inherent socio-economic patterns as well as types of facilities for supporting various urban services (reflected by POIs) are associated with the recovery. For the latter purpose, two deep learning models are adopted, including a CNN model and a Guided Grad-CAM model. These two models were built by making use of Keras and TensorFlow, and the implementations relied on pandas, numpy, sklearn, scipy, keras, shapely, rasterio and rasterstats python libraries.

(1) CNN model: To infer the urban recovery from aerial images, a CNN model^41^ was used. This model adopts the original EfficientNetB0 (EB0) architecture and appends a max pooling layer, followed by global average pooling and dense layers for: first learning from the actual urban recovery and then predicting probabilities of urban recovery levels (from one to five). EfficientNet models were built upon based on the following implementation: https://github.com/qubvel/efficientnet. EB0 is effective in terms of visualization learning performance because it supplies a sequence of mobile inverted bottleneck blocks (MBConv) augmented with squeeze-and-excitation optimization. Here the original EB0 model was trained on object-centric 224 × 224 × 3 images. And the model was trained by transfer learning from ImageNet and without freezing any layer. The input images were flipped randomly in both horizontal and vertical ways for augmenting the data and applying an Adam variant of stochastic gradient descent to learn the model weights. To improve the robustness of the model, a fivefold cross-validation was adopted in the training procedure, where, in each fold, 80% of the aerial images were split into a training set and the remaining 20% were withheld randomly. The training set was further randomly subdivided with 75–25% splits for inner-fold training and validation, and performance metrics were then averaged.

(2) Guided Grad-CAM: Following the last convolution layer of the above CNN model, the Guided Grad-CAM visualization algorithm was applied to explain and visualize the importance of visual attributes of aerial images detected by CNN. As a result, activation maps were first generated with highlighted features that were visualized based on pixels of aerial images. And then, activation maps and land use patterns were spatially combined for extracting important land use patterns associated with urban recovery.

### Spatial clustering of urban functions for the recovery: BiLISA

Influences of urban functions on the recovery at a certain area may have similar or dissimilar features observed at its neighboring areas. To better capture such spatial distributions, BiLISA is adopted with respect to two variables representing associations of urban functions with the recovery: i.e., activation rate and mean activation value, at the street-block level. An activation value of a certain urban function represents its importance of the function under study to the recovery level. The activation rate of a street-block is equal to the number of 0.49 m × 0.49 m pixels with an activation value being larger than zero, divided by the number of pixels within the corresponding street-block. The higher the activation rate, the more important the corresponding street-block in associating with urban recovery. The mean activation value for a street-block is equal to the sum of activation values (all values, including 0) of all 0.49 m × 0.49 m pixels, divided by the number of pixels within the street-block. A higher mean activation value for a street-block indicates that even though there are not many 0.49 m × 0.49 m pixels that are highly associated with the recovery, those highly associated pixels within the street-block may still play crucial roles in promoting the recovery. With respect to the influence of the mean activation value, it is necessary to identify what types of pixels having higher associations with the recovery. We assessed the BiLISA-derived clusters/outliers based on the minimum 999 permutations at the pseudo significance levels of 0.01 and 0.05, because the effect of the number of permutations is typically marginal relative to the value of 999. The formula of BiLISA can be found at the Section 3I in Supplementary Information.

## Supplementary Information

Supplementary Information.

## Data Availability

Concerning mobile phone signaling data, we purchased from NTT DOCOMO company, who does not allow us to share the data to anybody else due to both privacy protection and commercial restrictions. Land use data are available from “https://www.geospatial.jp/ckan/dataset/nagoya-kiso”. VIIRS night light satellite data are collected use Google Earth Engine (https://earthengine.google.com). Point of Interests (POIs) data can be obtained from the Esri Japan (https://www.esrij.com). The corresponding author will be responsible to answer any request on data.

## Abbreviations

CNN: Convolutional neural network
Guided Grad-CAM: Guided gradient-weighted class activation mapping
BiLISA: Bivariate local indicator of spatial association
GIS: Geographical information system
POIs: Points of interests
FiL: Field land
FoL: Forest land
RiL: River land
ONL: Other natural land
ReL: Residential land
CL: Commercial land
IL: Industrial land
PFL: Public facility land
RoL: Road land
TFL: Transportation facility land
POSL: Public open space
OPFL: Other public facility land
OOSL: Other open space land
IL_RoL: Industrial lands with road lands
PFL_ReL&RoL: Public facility lands with residential lands and road lands
FoL_ReL&RoL: Forest lands with residential lands and road lands
OPFL_ReL: Other public facility lands with residential lands
FiL_dm: Field lands dominated
RoL_ReL&CL: Road lands with residential lands and commercial lands
ReL_RoL: Residential lands and road lands
TFL_RoL: Transportation facility land with road lands
OPFL_RoL: Other public facility land with road lands
CL_ReL&RoL: Commercial lands with residential lands and road lands
OOSL_dm: Other open space lands dominated
