# Supplementary material for: Spatial and deep learning analyses of urban recovery from the impacts of COVID-19
Source: Sci Rep. 2023 Feb 11;13:2447. doi: 10.1038/s41598-023-29189-5 (PMC9922321; doi:10.1038/s41598-023-29189-5)
Supplement: Supplementary file 1 — Supplementary Information. [file 41598_2023_29189_MOESM1_ESM.docx]

**Supplementary Information for**

Spatial and deep learning analyses of urban recovery from the impacts of COVID-19

Shuang Ma^a,1^, Shuangjin Li^b,1^, Junyi Zhang^b,c,*^

^1^ **Joint first-author**: Equally contributed to this research

***Corresponding Authors:** Junyi Zhang

**Email:** zjy@hiroshima-u.ac.jp

**Data for research on urban recovery from COVID-19.** To measure the urban recovery level, the most widely applied data is probably human mobility data released by Google's COVID-19 Community Mobility Reports, followed by mobile phone signaling data. For instance, Romanillos et al. argued that mobile phone data have a great potential for analyzing the effectiveness of the restrictions on mobility for containing the COVID-19 spread (^[[1]](#endnote-1)^). Nomura et al. analyzed mobility data at 35 major stations and downtown areas in Japan to find new insights about human mobility and movement restrictions that are encouraged (but not forced) by the emergency declaration in Japan across age group (^[[2]](#endnote-2)^). Jia et al. analyzed how population flow from Wuhan, China influenced the spatiotemporal distribution of infected cases in China by using over 11 billion counts of mobile phone data on January 1–24, 2020 (^[[3]](#endnote-3)^). Chinazzi et al. examined the effect of international travel restrictions on mitigating the COVID-19 spread by adopting a global metapopulation disease transmission model based on data of Google's COVID-19 Community Mobility Reports (^[[4]](#endnote-4)^). The night-time light data were used to investigate the regional disparities and spatiotemporal changes of human urban socioeconomic activities in 20 global megacities before and during the COVID-19 pandemic (^[[5]](#endnote-5)^). Even though aerial images with 0.49m*0.49m resolution include rich information about urban functions at the facility level, no study has been found to apply such image data to reveal the urban recovery level.

**Countries/regions/cities targeted by research on urban recovery from COVID-19.** Globally, Huang et al. analyzed over 580 million tweets worldwide to investigate the global trends of human mobility by proposing a mobility-based responsive index (^[[6]](#endnote-6)^). At the country level, Chang et al. applied mobile phone data from 98 million people to examine the relationship between COVID-19 transmission and population movements to POIs at neighborhoods and proposed restrictions and reopening recommendations based on maximum occupancy at each POI in the US (^[[7]](#endnote-7)^). Lee et al. estimated the unequal recovery of employment of COVID-19 shock and found that demographic and social-economic attributes (e.g., gender, age, ethnicity, and education level) influenced the recovering process in the US using the monthly Current Population Survey from the Bureau of Labor Statistics (^[[8]](#endnote-8)^). Using 200K mobility data in Tokyo, Japan, Yabe et al. revealed an obvious inequality between different income groups in the sense that households in high-income regions were able to reduce social contacts, but households in low-income regions did not have such a flexibility (^[[9]](#endnote-9)^).

**2 Results**

**2A Spatial distributions of urban recovery levels**. Lower recovery levels were mainly observed at central areas in Nagoya, such as Higashi district (the recovery level was especially low for commercial activities), Naka district, Showa district, Atsuta district and Mizuho District. These districts had the highest population density and famous sightseeing sites and art centers, such as Nagoya Castle, Atsuta Shrine, and Aichi Museum of Art. Higher recovery levels appeared at peripheral areas of the city center and suburbs, where Moriyama district located at the north-east corner had the lowest population density among all 16 districts in Nagoya but its recovery level was very high.

**2B Estimating and validating an EfficientNetB0-based urban recovery prediction model.** As for the matrices in the Fig. 1 *g*-*i* in the main text, the smaller the deviation from the diagonal, the greater the prediction accuracy. Three accuracy indicators were calculated: i.e., accuracy (ACC), mean average error (MAE), and Pearson correlation coefficient (PCC), with respect to actual urban recovery levels and predicted ones. The ACCs were between 0.41 (working activity) and 0.35 (commercial activity). The ACC refers to the proportion of samples with correct predictions in all samples. It has limitations in reflecting the performance of the model when the dataset classifications are unbalanced. Therefore, the other two accuracy indicators were also chosen. The PCC values for the selected three activities are 0.684, 0.632 and 0.589, respectively, while the corresponding MAE values are all 1.0.

**2C Associating aerial images and “land use and pre-pandemic socio-economic activity” patterns with urban recovery through activation maps.** Fig. S1 (following Fig. 2 *a*-*e* in the main text) shows activation rates and mean activation values at different street-blocks in a mesh. Higher activation rate and mean activation value indicate the importance degree of a certain land use and social-economic pattern to the urban recovery.

**3 Materials and Methods**

**3A Study area.** Nagoya (Fig. S2) is the fourth most populous city (2.3 million) in Japan. The first death of COVID-19 infection was confirmed in Japan on February 13, 2020 and the Japanese government announced to close primary, secondary and high schools from March 2 to April 6, 2020. From April 10 to May 25, 2020, the Nagoya government declared a state of emergency for the first time to prevent the further spread of COVID-19. This declaration led to zero daily infection in Nagoya on May 15, 2020, lasting for 23 days. In this study, the first week after the emergency declaration, i.e., between May 26 (Tuesday) and June 1 (Monday) in 2020, when there were no additional infections, was selected to represent a recovery period, which was compared with the same week between May 28 (Tuesday) and June 3 (Monday) in 2019.

**3B Data. Mobile phone signaling data.** Mobile phone signaling data can record spatiotemporal human activity trajectories. The data were obtained from NTT DOCOMO company, which is the largest mobile phone company in Japan. This company had 82.63 million mobile phone users in March 2021, accounting for 43.8% of the mobile phone market in Japan. Original point-to-point data were aggregated into 500m*500m meshes for being released to the public, where the total number of population in each mesh is calculated at the hour level. There are a total of 1,383 meshes in Nagoya, covering an area of 345.75 km^2^.

**3C Data. Aerial images.** High-resolution satellite images of Nagoya in 2019 were obtained from Google Static Maps. The zoom of each image is set at Level 18, which corresponds to a resolution of 0.49m x 0.49m. Satellite images were clipped as a series of georeferenced 500m x 500m tiles, each of which corresponds to a mobile phone signaling square mesh in the ArcGIS Pro platform. The various visual attributes, captured by a certain number of layers with color, shade, shape, and texture and so on that can indirectly represent urban functions, are used to infer the urban recovery based on deep learning models.

**3D Data. Land use data.** Land use data were obtained from the City Planning Basic Survey of Land Use in Nagoya in 2017 (<https://www.geospatial.jp/ckan/dataset/nagoya-kiso>), covering 4,086 street-blocks over the whole city. This data set includes percentages of 13 types of land use at the street-block level: field land (FiL), forest land (FoL), river land (RiL), other natural land (ONL), residential land (ReL), commercial land (CL), industrial land (IL), public facility land (PFL), road land (RoL), transportation facility land (TFL), public open space (POSL), other public facility land (OPFL) and other open space land (OOSL).

**3E Data. VIIRS night light satellite data.** Night time light (NTL) data represents the distributions of artificial nocturnal radiances across human settlements. It has been widely used to measure the level of socio-economic activities (^[[10]](#endnote-10)^-^[[11]](#endnote-11)^). In this study, monthly-averaged radiance composite images were collected during January-December in 2019 from the dataset of “VIIRS Stray Light Corrected Night Time Day/Night Band (DNB) Composites Version 1” in Nagoya by Google Earth Engine (<https://earthengine.google.com>) with a higher spatial resolution (15 arc—seconds, which is around 500m x 500m) and wider quantization range (14 bits). The data processing includes removing the background noise and temporal fires filtration. To denoise the data, if the night radiance has a negative value, then a radiance value 0 was set. The temporal fires are shorter and happen at various locations, where they are filtered through an eight-connected-component labeling method (^[[12]](#endnote-12)^-^[[13]](#endnote-13)^). The average NTL radiance value reflects the intensity of social-economic activities. Based on the average values, all the 4,086 street-blocks were divided into three types: i.e., street-blocks with high-level, medium-level and low-level social-economic activities, based on a quantile method. These three types are used to reflect the pre-pandemic socio-economic activity patterns. Because the spatial resolution of NTL can be larger than the street-block level, the street-blocks at the same NTL mesh are assigned with a same value.

**3F Data. Point of Interests (POIs) data.** There are 39,761 POIs from 263 categories in Nagoya, as shown in data from the 2018 ESRI Japan Corporation. POIs data were converted into 16 large categories: tourism attraction, transportation service, governmental & social group, culture & education, medical service, science/culture service, sports & recreation, food & beverages, shopping, auto sale, auto service, finance service, daily life service, accommodation service, service apartment, and enterprise. Each POI corresponds to a 0.49m*0.49m pixel. To figure out the important POIs for the urban recovery, those POIs with an activation value within the top one-third of all activation values were selected.

**3G Measurement of urban recovery.** The urban recovery from COVID-19 was measured with respect to the three types of activities at the mesh level, where the main time slots of each day were selected: i.e., 09:00–17:00 on Monday–Friday for working activities, 18:00–24:00 on Monday–Friday for night-life activities, and 09:00–24:00 on Saturday–Sunday for commercial activities. Furthermore, because of stay-at-home interventions, many people had to spend longer time at home during the COVID-19 pandemic than the pre-pandemic period. It is therefore not proper to make use of activities performed at residential areas to measure the urban recovery. Thus, the meshes with main functions of residence were excluded from the data used in this study (1,383 meshes). As a result, more than 50% of such meshes were excluded and the remaining 1209 meshes were left to evaluate the urban recovery in this study. For ease of capturing changes in various activities, the above-calculated levels of urban recovery were further partitioned into five categories from low to high by using a five-quantile method to guarantee that each category has the same number of meshes.

**3H Categorizing land use patterns based on Elbow and K-means.** In order to categorize all the 4,086 street-blocks based on percentages of different land uses, the Elbow method in Python was first applied. A turning point in the trend of the sum of the squared errors (SSE) determines whether there are large gaps between each sample and all other samples within the same cluster or across different clusters (^[[14]](#endnote-14)^). The turning point was observed when the number of clusters reaches 12, meaning that all the street-blocks can be divided into 12 patterns. Furthermore, based on the sum of the squared errors calculated in the K-means method and by inputting the number of patterns into SPSS software, all the street-blocks were divided into 12 patterns by reflecting the features of percentages of different land use types. This can objectively determine the number of patterns, rather than subjectively putting any pattern number in K-means method in SPSS software and deciding the final pattern number by comparing the results of specific classifications for each pattern number. The name and distribution of the number of land use patterns for the total 4,086 street-blocks are illustrated in Fig. S3.

**3I Spatial clustering of urban functions for the recovery: BiLISA.** The formula of BiLISA can be defined as:

$$I_{i,kl}=Z_{i,k}\sum_{j=1}^{N} w_{ij}Z_{j,l}$$

where $Z_{i,k}=(X_{i,k}-X_{k,mean})/\sigma_{k}$, $Z_{j,l}=(X_{j,l}-X_{l,mean})/l$; *N* refers to the number of all the street blocks in Nagoya under study; *Xi*,*k* and *Xj*,*l* indicate the activation rate in spatial unit (street block) *i* and the mean activation value in its neighboring street block *j* ( *j* $\neq$ *i*): the average values of these two variables are *Xk*,*mean* and *Xl*,*mean*, and their standard deviations are σ*k* and σ*l*, respectively; and *wij* measures the spatial weight between spatial units *i* and *j*. The significance of the BiLISA statistic $I_{i,kl}$ is evaluated at the 95% level based on randomization permutations, which can effectively reduce uncertainties (^[[15]](#endnote-15)^).


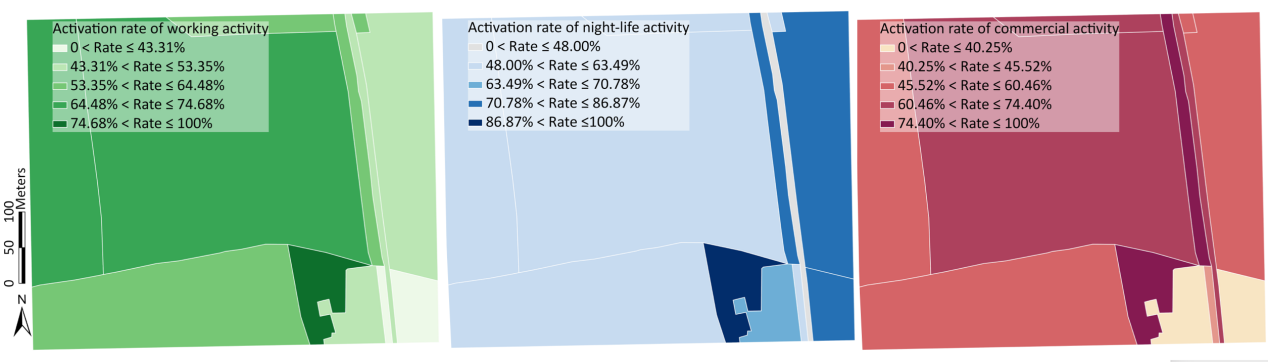


*a*. Activation rates for *b*. Activation rates for *c*. Activation rates for

working activity night-life activity commercial activity

***
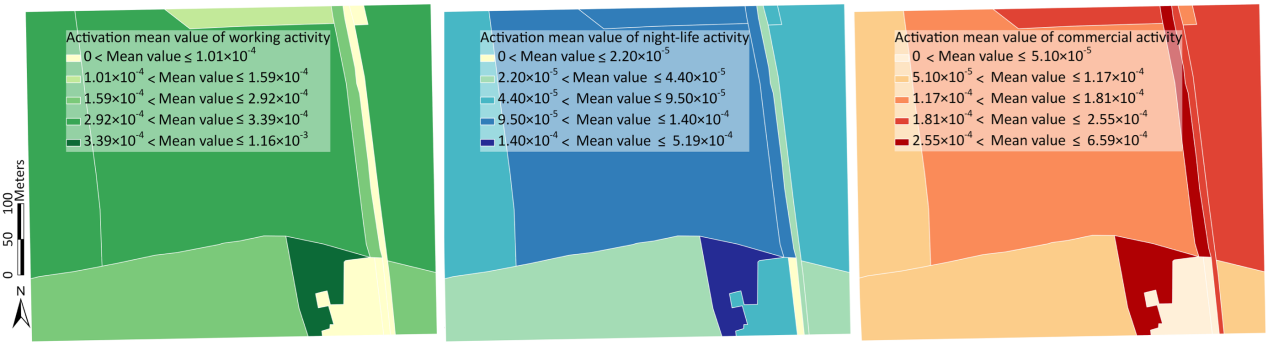
***

*d*. Activation means value for *e*. Activation means value for *f*. Activation means value for

working activity night-life activity commercial activity

**Fig. S1** Activation rates and mean activation values at different street blocks in the mesh example shown in Fig. 2 *a*-*e*


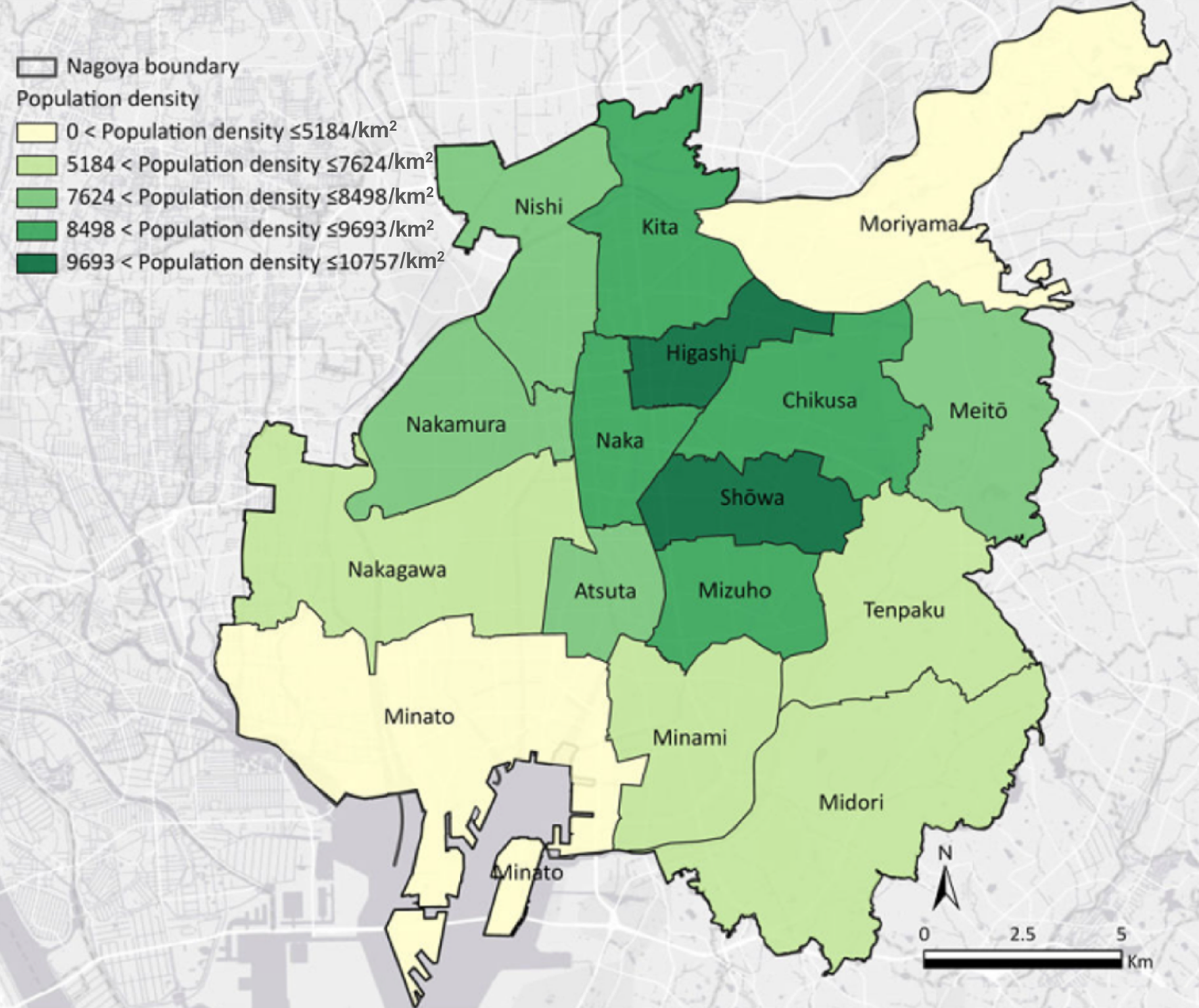


**Fig. S2** Spatial distributions of population density in Nagoya, Japan. Created using ArcGIS Pro Version 2.9 from ESRI (http://www.arcgis.com/).


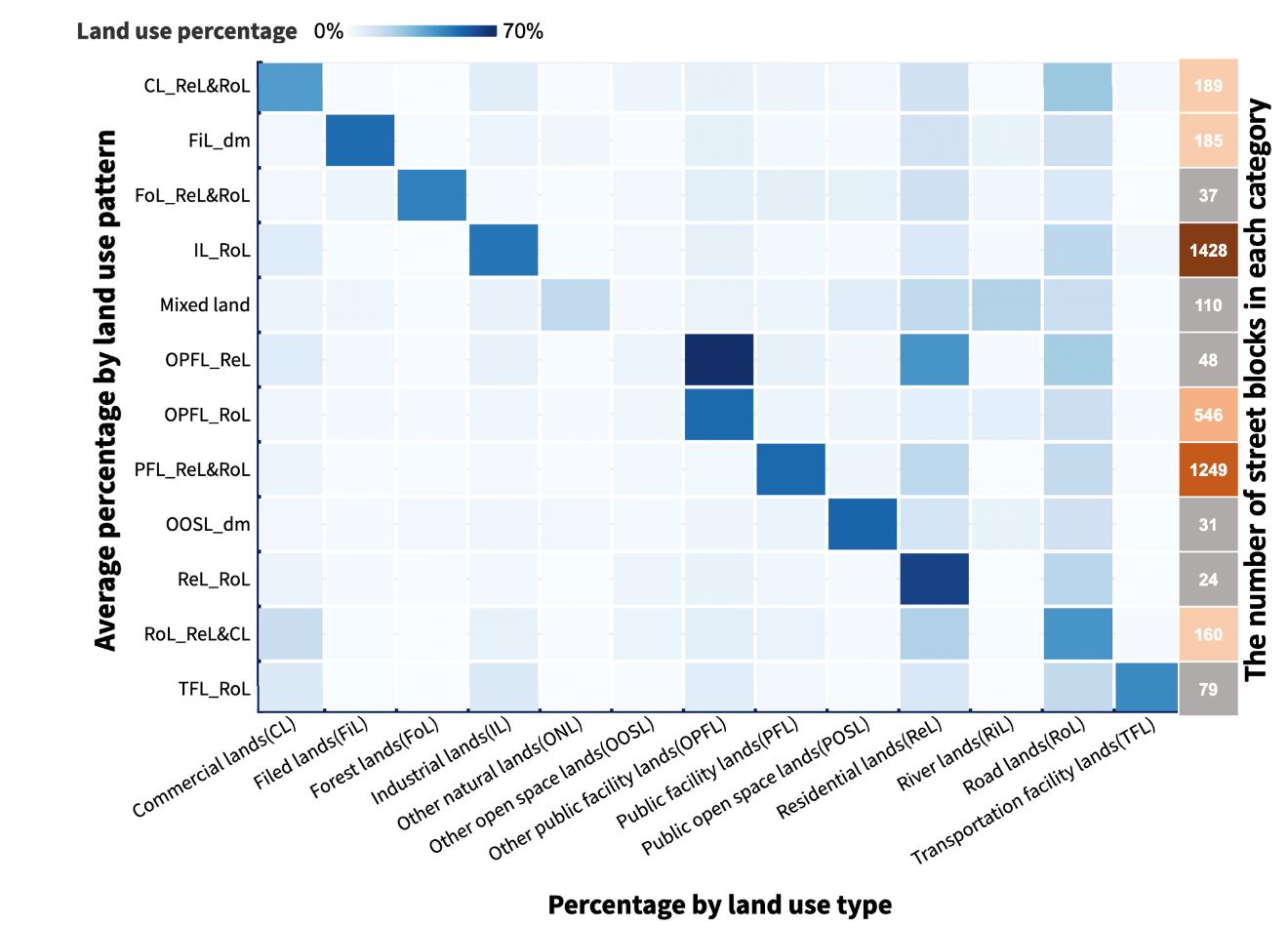


**Fig. S3** The derived land use patterns through Elbow method and K-means method. Created using Flourish platform (https://flourish.studio).

*[Note] IL_RoL: Industrial lands with road lands; PFL_ReL&RoL: public facility lands with residential lands and road lands; FoL_ReL&RoL: forest lands with residential lands and road lands; OPFL_ReL: other public facility lands with residential lands; FiL_dm: field lands dominated; RoL_ReL&CL: road lands with residential lands and commercial lands; ReL_RoL: residential lands and road lands; TFL_RoL: transportation facility land with road lands; OPFL_RoL: other public facility land with road lands; CL_ReL&RoL: commercial lands with residential lands and road lands; OOSL_dm: other open space lands dominated.*

**SI References**

1. Romanillos, G., *et al.* The city turned off: Urban dynamics during the COVID-19 pandemic based on mobile phone data. *Appl. Geogr.* **9**, 102524 (2021). [↑](#endnote-ref-1)
2. Nomura, S., *et al.* Mobility patterns in different age groups in Japan during the COVID-19 pandemic: a small area time series analysis through March 2021. *J. Urban Health* **98**, 635–641 (2021). [↑](#endnote-ref-2)
3. Jia, J., *et al.* Population flow drives spatio-temporal distribution of COVID-19 in China. *Nature* **582**, 389–394 (2020). [↑](#endnote-ref-3)
4. Chinazzi, M., *et al.* The effect of travel restrictions on the spread of the 2019 novel coronavirus (COVID-19) outbreak. *Science* **368**, 395–400 (2020). [↑](#endnote-ref-4)
5. Xu, G., *et al.* Lockdown induced night-time light dynamics during the COVID-19 epidemic in global megacities. *Int. J. Appl. Earth Obs. Geoinf* **102**, 02421(2021). [↑](#endnote-ref-5)
6. Huang, X. *et al.,* Twitter reveals human mobility dynamics during the COVID-19 pandemic. *Plos One* **15**, e0241957 (2020). [↑](#endnote-ref-6)
7. Chang, S., *et al.* Mobility network models of COVID-19 explain inequities and inform reopening. *Nature* **589**, 82–87 (2021). [↑](#endnote-ref-7)
8. Lee, S. Y., Park, M. & Shin, Y. Hit harder, recover slower? Unequal employment effects of the COVID-19 shock. *Review* **103**, 4 (2021). [↑](#endnote-ref-8)
9. Yabe, T., *et al.,* Non-compulsory measures sufficiently reduced human mobility in Tokyo during the COVID-19 epidemic. *Sci. Rep.* **10**, 1–9 (2020). [↑](#endnote-ref-9)
10. Falchi, F., *et al.*, The new world atlas of artificial night sky brightness. *Sci. Adv.* **2**, e1600377 (2016). [↑](#endnote-ref-10)
11. Elvidge, C. D., Hsu, F. C., Baugh, K. E. & Ghosh, T. National trends in satellite observed lighting: 1992–2012. *Global urban monitoring and assessment through earth observation*. CRC Press (2014). [↑](#endnote-ref-11)
12. Ma, S., Kumakoshi, Y., Koizumi, H. & Yoshimura, Y. Discovering the association of the built environment and socioeconomic factors with urban shrinking in Yokohama City. *Cities* **120**, 103474 (2021). [↑](#endnote-ref-12)
13. Yuan, X., Jia, L., Zhou, J., Menenti, M. & Chen, Q. A new method for noise removal in Npp-Viirs monthly nighttime light imagery over the Sahel region. *IEEE Int. Symp. on Geosci. Remote Sens.* 7467–7470 (2019). [↑](#endnote-ref-13)
14. Aytaç, E. Unsupervised learning approach in defining the similarity of catchments: Hydrological response unit based k-means clustering, a demonstration on Western Black Sea Region of Turkey. *Int. Soil Water Conserv. Res.* **8**, 321–331 (2019). [↑](#endnote-ref-14)
15. Anselin, L. Local indicators of spatial association—LISA. *Geogr. Anal.* **27**, 93–115 (1995). [↑](#endnote-ref-15)
